# Ceramide compensation by ceramide synthases preserves retinal function and structure in a retinal dystrophy mouse model

**DOI:** 10.1242/dmm.050168

**Published:** 2023-07-19

**Authors:** Xinye Qian, Tanmay Srinivasan, Jessica He, Jiaxiong Lu, Yan Jin, Haiwei Gu, Rui Chen

**Affiliations:** ^1^ Verna and Marrs McLean Department of Biochemistry and Molecular Biology, Baylor College of Medicine, Houston, TX 77030, USA; ^2^ Human Genome Sequencing Center, Baylor College of Medicine, Houston, TX 77030, USA; ^3^ Rice University, Houston, TX 77030, USA; ^4^ Center for Translational Science, Florida International University, Port St. Lucie, FL 34987, USA; ^5^ Department of Molecular and Human Genetics, Baylor College of Medicine, Houston, TX 77030, USA

**Keywords:** Inherited retinal dystrophy, TLCD3B, Ceramide, Ceramide synthase, Retina

## Abstract

Increasing evidence has supported the role of ceramide as a mediator of photoreceptor dysfunction or cell death in ceramide accumulation and deficiency contexts. TLCD3B, a non-canonical ceramide synthase, was previously identified in addition to the six canonical ceramide synthases (CerSs), and the *Tlcd3b^−/−^* mouse model exhibited both retinal dysfunction and degeneration. As previous canonical CerS-deficient mouse models failed to display retinal degeneration, the mechanisms of how TLCD3B interacts with CerSs have not been investigated. Additionally, as the ceramide profile of each CerS is distinct, it is unclear whether the overall level or the homeostasis of different ceramide species plays a critical role in photoreceptor degeneration. Interactions between TLCD3B with canonical CerSs expressed in the retina were examined by subretinally injecting recombinant adeno-associated virus 8 vectors containing the *Cers2* (*rAAV8-CerS2*), *Cers4* (*rAAV8-CerS4*) and *Cers5* (*rAAV8-CerS5*) genes. Injection of all three *rAAV8-CerS* vectors restored retinal functions as indicated by improved electroretinogram responses, but only *rAAV8-CerS5* successfully retained retinal morphology in *Tlcd3b^−/−^* mice. CerSs and TLCD3B played partially redundant roles. Additionally, rather than acting as an integral entity, different ceramide species had different impacts on retinal cells, suggesting that the maintenance of the overall ceramide profile is critical for retinal function.

## Teaser

**Summary:** Overexpression of ceramide synthases compensated ceramide levels in a retinal dystrophy mouse model. Different ceramide species had different impacts on retinal cells, highlighting the importance of maintaining overall ceramide profile for retinal functions.

## INTRODUCTION

Ceramide, a class of sphingolipids abundant in cell membranes, is a bioactive lipid that functions as an important cellular second messenger that signals for apoptosis (Hannun, 1996; Hannun and Obeid, 2002; Pettus et al., 2002). As the central molecule of the highly interconnected sphingolipid metabolic pathways, ceramide forms the backbone of all complex sphingolipids and is an essential player in key cellular functions, including the regulation of cell-stress responses (Scarlatti et al., 2004; Hannun and Obeid, 2008), the modulation of membrane dynamics (Holopainen et al., 2000; Stancevic and Kolesnick, 2010) and the activation of cellular pathways by acting as a second messenger (Chalfant et al., 1999; Ruvolo, 2003; Wang et al., 2005; Canals et al., 2018). In mammals, *de novo* ceramide synthesis is catalyzed by six ceramide synthases (CerSs): CerS1-6 (Mullen et al., 2012). Each CerS regulates the formation of a specific set of ceramides with variable chain lengths and mediates distinct functions (Mizutani et al., 2005; Imgrund et al., 2009; Levy and Futerman, 2010; Ginkel et al., 2012; Jennemann et al., 2012; Ebel et al., 2014). Specifically, CerS1 synthesizes mainly C18 ceramide, CerS2 synthesizes mainly C22-C24 ceramide, CerS3 synthesizes very long chain ceramides (>C26), CerS4 synthesizes C18-C20 ceramide, and CerS5 and CerS6 synthesize mostly C16 ceramide (Ben-David et al., 2011). Consequently, there is redundancy in the ceramides synthesized by each synthase, but no CerS can completely compensate for the function of another. CerSs demonstrate tissue-specific expressions, which lead to ceramide profile variations among different tissues (Levy and Futerman, 2010). Ceramides with different chain lengths have various biological functions, and ceramide imbalance may trigger stress responses or cause diseases. *Cers1*-deficient mice exhibited cerebellar shrinkage and Purkinje cell loss (Ginkel et al., 2012), whereas CerS2 was essential for the production of myelin by oligodendrocytes (Ben-David et al., 2011). The production of very-long-chain (>C26) ceramides by CerS3 appears to be essential in the maintenance of a functional epidermal barrier (Mizutani et al., 2008, 2009). *Cers4*-deficient mice, however, suffer from progressive hair loss (Ebel et al., 2014). As a result of the interplay among CerSs, the homeostasis of different ceramide species also has a profound impact. In *Cers1*-deficient mice, despite the overall ceramide level reduction, the dramatic decrease in C18 ceramides triggered the overproduction of C16 ceramides, which failed to compensate for the damage caused by C18 deficiency (Zhao et al., 2011). In *Cers2*-deficient mice, C24:0 and C24:1 ceramides declined in association with a compensatory increase in C16 and C18 ceramides. However, the total level of ceramides was unaltered, suggesting the distinct roles played by ceramide species with variable chain lengths (Ben-David et al., 2011).

Increasing evidence supports the role of ceramide as a mediator of photoreceptor death, which is integral to major inherited retinal diseases including retinitis pigmentosa, Stargardt's disease, Leber's congenital amaurosis and age-related macular degeneration (Glazer and Dryja, 2002; Allikmets, 2004; Haddad et al., 2006; Hartong et al., 2006). On the one hand, retinal cell death or retinal impairment accompanied by ceramide accumulation has been observed both *in vivo* (Acharya et al., 2003, 2008; Ranty et al., 2009; Strettoi et al., 2010) and *in vitro* (German et al., 2006; Sanvicens and Cotter, 2006; Chen et al., 2012). On the other hand, ceramide deficiency has also recently been associated with retinal dysfunction and degeneration, as evidenced by the deleterious effects on the retina owing to ceramide depletion caused by CerS deletion (Brüggen et al., 2016; Bertrand et al., 2021; Qian et al., 2022). Retinal functions were previously examined in *Cers1*-, *Cers2*- and *Cers4*-deficient mice (Brüggen et al., 2016). Accompanied by the C20-C24 ceramide increase and the C16 ceramide reduction, retinal dysfunction was detected by electroretinograms (ERGs), but no significant morphological defects were detected in these three mouse models (Brüggen et al., 2016).

In addition to the canonical CerSs, TLC domain-containing protein 3B (TLCD3B) is also found to have CerS activity (Yamashita-Sugahara et al., 2013). Relative to the other six canonical CerSs in the retina, TLCD3B has a much higher expression level (Bertrand et al., 2021). Patients diagnosed with cone-rod dystrophy or maculopathy were found to have *TLCD3B* mutations, and their phenotypes were restricted to the retina (Bertrand et al., 2021). *Tlcd3b^−/−^* mice demonstrated both retinal dysfunction and degeneration by 7 months of age (Bertrand et al., 2021). TLCD3B has a distinct ceramide profile consisting of C16, C18 and C20 ceramides, which do not fully overlap with any of the six canonical CerSs (Yamashita-Sugahara et al., 2013; Qian et al., 2022). Although these results further support the importance of maintaining ceramide homeostasis in the retina, mechanistic explanations of the observed retinal morphological defects caused by TLCD3B disruption are still lacking. Given that previous canonical CerS-deficient mouse models failed to display retinal degeneration (Brüggen et al., 2016), an interesting question was brought up regarding the relationship between ceramide homeostasis and retinal cell death in the context of TLCD3B deficiency: is TLCD3B deficiency-associated retinal cell death caused by an absolute change in ceramide level, or does a healthy retina rely more on the profile maintenance of different ceramide species with variable chain lengths?

In a previous study (Qian et al., 2022), we performed gene therapy on *Tlcd3b^−/−^* mice and successfully rescued the retinal degeneration phenotypes by subretinally injecting a recombinant adeno-associated virus 8 (rAAV8) vector containing the *Tlcd3b* gene (*rAAV8-Tlcd3b*). Based on targeted metabolomics analyses, *Tlcd3b^−/−^* mice had a significant reduction of C16, C18 and C20 ceramides and the gene therapy successfully recovered the levels of these ceramide species, providing the first *in vivo* evidence that TLCD3B plays a role in ceramide production. To further explore the roles of TLCD3B and its interaction with canonical CerSs, we examined whether ceramide compensation in *Tlcd3b^−/−^* mice could rescue photoreceptor degeneration by performing subretinal injections of three adeno-associated-virus (AAV)-mediated canonical CerS genes (*rAAV8-CerS2*, *rAAV8-CerS4* and *rAAV8-CerS5*). To achieve high transduction efficiency, more rapid transgene expression, cell transduction specificity, and stable and long-term expression in photoreceptor cells, a tyrosine-capsid mutant AAV plasmid, AAV8 (Y733F) was used (Petrs-Silva et al., 2009; Ku et al., 2011; Pang et al., 2011; Zhong et al., 2015; Zaneveld et al., 2019). Although treatment with all three *rAAV8-CerS* vectors was able to restore retinal functions as indicated by improved ERG responses, only *rAAV8-CerS5* treatment successfully retained retinal morphology in *Tlcd3b^−/−^* mice. Additionally, mass spectrometry data and targeted metabolomics analyses suggested that *rAAV8-CerS5* treatment best recovered key ceramide species that are largely reduced in *Tlcd3b^−/−^* mice (C16:0, C18:0 and C20:0 ceramides) compared to the ceramide profiles of the other two viruses, indicating that different ceramide species may play different roles in retinal homeostasis. In summary, our results suggest that ceramide is a primary activator of retinal degeneration in *Tlcd3b^−/−^* mice. Moreover, rather than acting together, different ceramide species have different impacts on retinal cells, making maintenance of the overall ceramide profile essential to retinal cell functions.

## RESULTS

### *rAAV8*-mediated CerS gene transduction in photoreceptor cells

Given that the primary defect caused by loss of TLCD3B is mainly photoreceptor degeneration, we cloned mouse CerS cDNAs under the control of the human rhodopsin kinase (*hGRK1*) promotor and packaged the construct in an rAAV8 vector to achieve targeted expression in photoreceptor cells. During cloning, a sequence encoding an N-terminal FLAG tag was included in the vector to detect the expression of *rAAV8-CerS* vectors (Fig. S1). For the three treatment groups, *rAAV8-CerS2*, *rAAV8-CerS4* and *rAAV8-CerS5*, subretinal injections were performed at postnatal day (P)21 to deliver *rAAV8-CerS* vectors to the right eyes (REs) of *Tlcd3b^−/−^* mice, whereas the contralateral left eyes (LEs) were injected with PBS solutions to serve as internal controls.

**Fig. 1.**
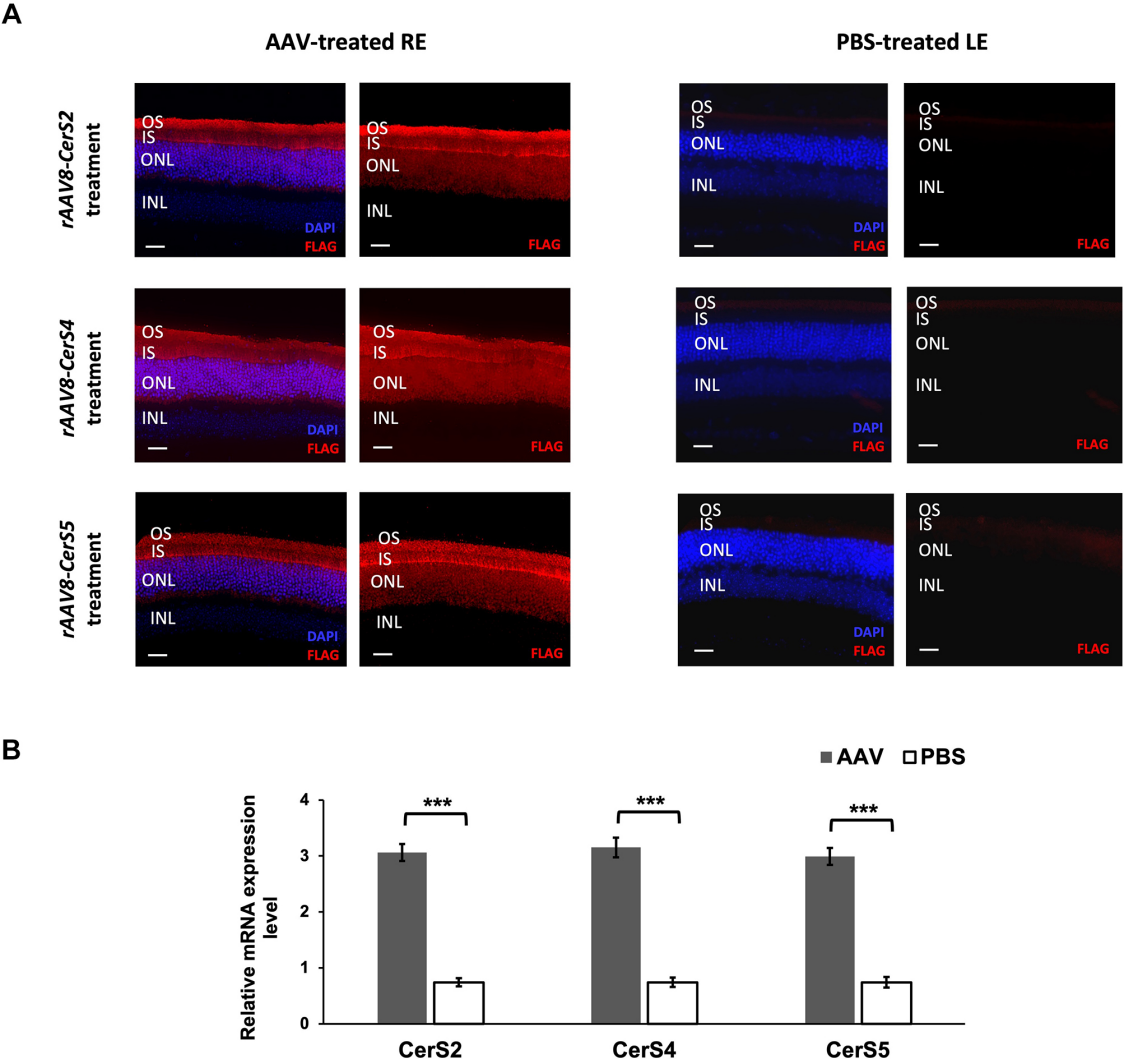
***rAAV8-CerS* expression in the treated mouse retinas and localization in the photoreceptor cells.** (A) Immunofluorescence staining of representative contralateral PBS-treated left eyes (LEs) and AAV-treated right eyes (REs) of 7-month-old *Tlcd3b^−/−^* mice that were subretinally injected with *rAAV8-CerS2*, *rAAV8-CerS4* or *rAAV8-CerS5* at postnatal day 21 (P21). FLAG signals (red), indicating CerS gene expression, were detectable only in the REs of *Tlcd3b^−/−^* mice, which demonstrated robust staining throughout different layers of photoreceptor cells, whereas the expression was undetectable in the LEs. DAPI staining was performed to stain cell nuclei. INL, inner nuclear layer; IS, inner segment; ONL, outer nuclear layer; OPL, outer plexiform layer. Images are representative of *n*=3 mice for each treatment group. Scale bars: 50 μm. (B) Higher CerS expression was detected in AAV-treated eyes (*n*=3) compared to PBS-treated eyes (*n*=3) by qPCR in 7-month-old mice. For each retina, qPCR was performed in triplicate. Error bars denote the s.e.m. Two-tailed unpaired Student’s *t*-tests were used to compare the relative mRNA expression. ***P<0.001.

The expression of *rAAV8-CerS* vectors was examined by immunofluorescence staining of the FLAG tag when mice were 7 months of age. In the treated REs of *Tlcd3b^−/−^* mice, for all three *rAAV8-CerS*s, positive FLAG-tagged transgene expression was robustly detected in photoreceptor cells, as indicated by universal expression across outer segments, inner segments and outer nuclear layers (ONLs) near the injection site (Fig. 1A). In contrast, in the PBS-injected LEs, no FLAG tag expression was observed. Additionally, we also performed quantitative PCR (qPCR) to examine CerS gene expression levels, and there were significantly higher levels of *Cers2*, *Cers4* and *Cers5* mRNAs in the treated eyes (Fig. 1B). Therefore, the subretinal injections of *rAAV8-CerS2*, *rAAV8-CerS4* and *rAAV8-CerS5* vectors all resulted in stable and efficient transgene expression in photoreceptor cells.

### Preservation of photoreceptor function by the overexpression of canonical CerS genes in *Tlcd3b^−/−^* mice

Based on previous studies, at 7 months of age, *Tlcd3b^−/−^* mice exhibited significant bipolar cell and cone photoreceptor photoresponse impairment (Bertrand et al., 2021). To determine the functional rescue by canonical CerSs, we examined electrophysiological responses to light in *Tlcd3b^−/−^* mice by conducting ERG tests. For the three canonical CerSs that were tested, three different dosages (high, medium and low) were examined for each treatment group, and ERG results demonstrated a clear dosage-dependent pattern (Fig. S2).

**Fig. 2.**
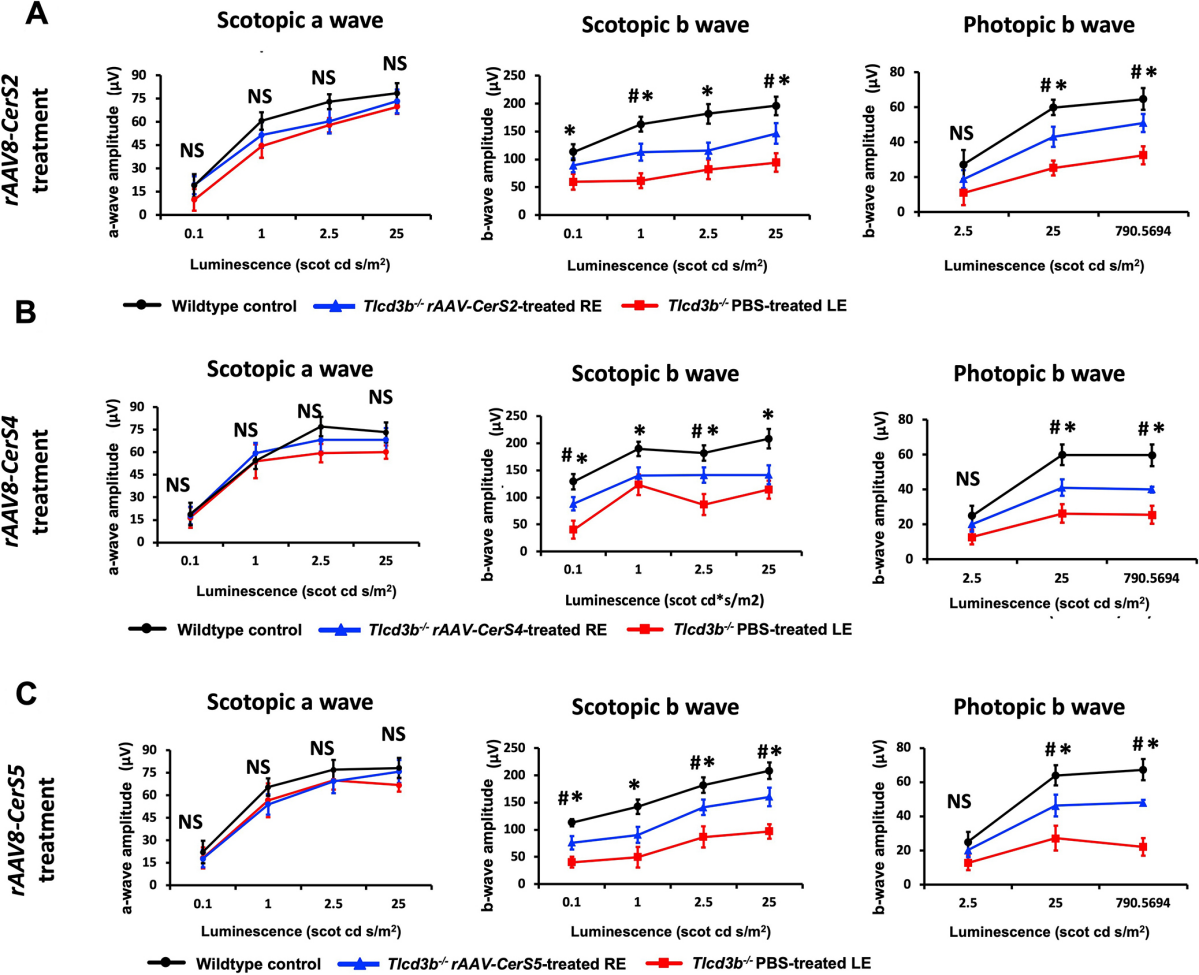
**Retinal photoresponses in *Tlcd3b^−/−^* mice were rescued by canonical CerSs.** (A-C) Quantitative evaluation of full-field electroretinography was performed on 7-month-old dark-adapted *Tlcd3b^−/−^* mice that were treated by subretinal injections of *rAAV8-CerS2* at a dosage of 1.38×10^11^ gc/ml (A), *rAAV8-CerS4* at a dosage of 1.03×10^10^ gc/ml (B) and *rAAV8-CerS5* at a dosage of 7.5×10^11^ gc/ml at P21 (C). A two-way repeated measures ANOVA with Sidak post hoc test for multiple comparisons was performed. *rAAV8-CerS2* led to a 57% improvement (*P*=0.01; 68% of wild type) of scotopic b-wave responses and 76% improvement (*P*=0.005; 72% of wild type) of photopic b wave responses in REs compared to LEs. *rAAV8-CerS4* led to a 57% improvement (*P*=0.02; 66% of wild type) of scotopic b-wave responses and 54% improvement (*P*=0.009; 62% of wild type) of photopic b-wave responses in REs compared to LEs. *rAAV8-CerS5* led to a 74% improvement (*P*=0.007; 74% of wild type) of scotopic b-wave responses and 81% improvement (*P*=0.001; 75% of wild type) of photopic b-wave responses in REs compared to LEs. *n*=6 mice were used to assay LE and RE ERGs. *n*=4 wild-type mice were used as technical controls. Two-tailed unpaired Student’s *t*-tests were performed at each luminescence. Statistical analysis is denoted with the following symbols: NS, not significant; wild type versus untreated, *P<0.05; untreated versus treated, ^#^*P*<0.05. Error bars denote the s.e.m. scot, scotopic matched.

For *rAAV8-CerS2*, the medium dosage of 1.38×10^11^ genome copies (gc)/ml successfully preserved retinal functions of REs compared to those of LEs in *Tlcd3b^−/−^* mice. Specifically, there was a functional improvement in cone photoreceptor cells and phototransduction/inner retinal neuron response, as indicated by significantly enhanced scotopic and photopic b-wave amplitudes of REs (Fig. 2A). However, scotopic a-waves of REs were not affected, indicating that the medium dosage of *rAAV8-CerS2* does not impair rod photoreceptor function. Although the low dosage of 1.38×10^10^ gc/ml showed neither beneficial nor detrimental impact on retinal functions, the high dosage of 1.38×10^12^ gc/ml largely impaired photoresponses, as indicated by significantly reduced scotopic a-wave, and scotopic and photopic b-wave amplitudes (Fig. S2A).

*rAAV8-CerS4*, however, demonstrated rescue potential at the low dosage of 1.03×10^10^ gc/ml (Fig. 2B). AAV-treated REs showed significantly enhanced scotopic and photopic b-waves compared to PBS-treated LEs, suggesting successful preservation of cone photoreceptor cell and bipolar cell functions or phototransduction by low-dosage *rAAV8-CerS4* treatment. Although the high dosage of *rAAV8-CerS4* also resulted in impaired retinal functions at a dosage of 1.03×10^12^ gc/ml, the medium dosage of 1.03×10^11^ gc/ml did not have any significant impact on the REs of *Tlcd3b^−/−^* mice compared to the LEs (Fig. S2B).

*rAAV8-CerS5* demonstrated a similar dosage-dependent pattern to that of *rAAV8-CerS2*. The medium dosage of 7.5×10^11^ gc/ml significantly improved scotopic and photopic b-wave amplitudes in AAV-injected REs compared to those of PBS-injected LEs (Fig. 2C). Specifically, *rAAV8-CerS5* achieved the best functional recovery of cone cells compared to that achieved by the other two viruses (Fig. S2). Scotopic a-wave responses in REs did not show any significant difference compared to those in LEs, suggesting intact rod photoreceptor functions. Similar to *rAAV8-CerS2*, *rAAV8-CerS5* also showed a detrimental impact on retinal functions at the high dosage of 7.5×10^12^ gc/ml and no impact at the low dosage of 7.5×10^10^ gc/ml (Fig. S2C).

Collectively, at their functional rescue dosages, *rAAV8-CerS5* demonstrated the best rescue potential, followed by *rAAV8-CerS2* and *rAAV8-CerS4* (Fig. 2), as indicated by the 75% improvement of scotopic b-wave responses (80% of those of wild type) and 82%improvement of photopic b-wave responses (79% of those of wild type) in REs compared to LEs in *rAAV8-CerS5-*treated mice.

### The delivery of *rAAV8-CerS5* successfully preserved photoreceptor integrity in *Tlcd3b^−/−^* mice

**Fig. 3.**
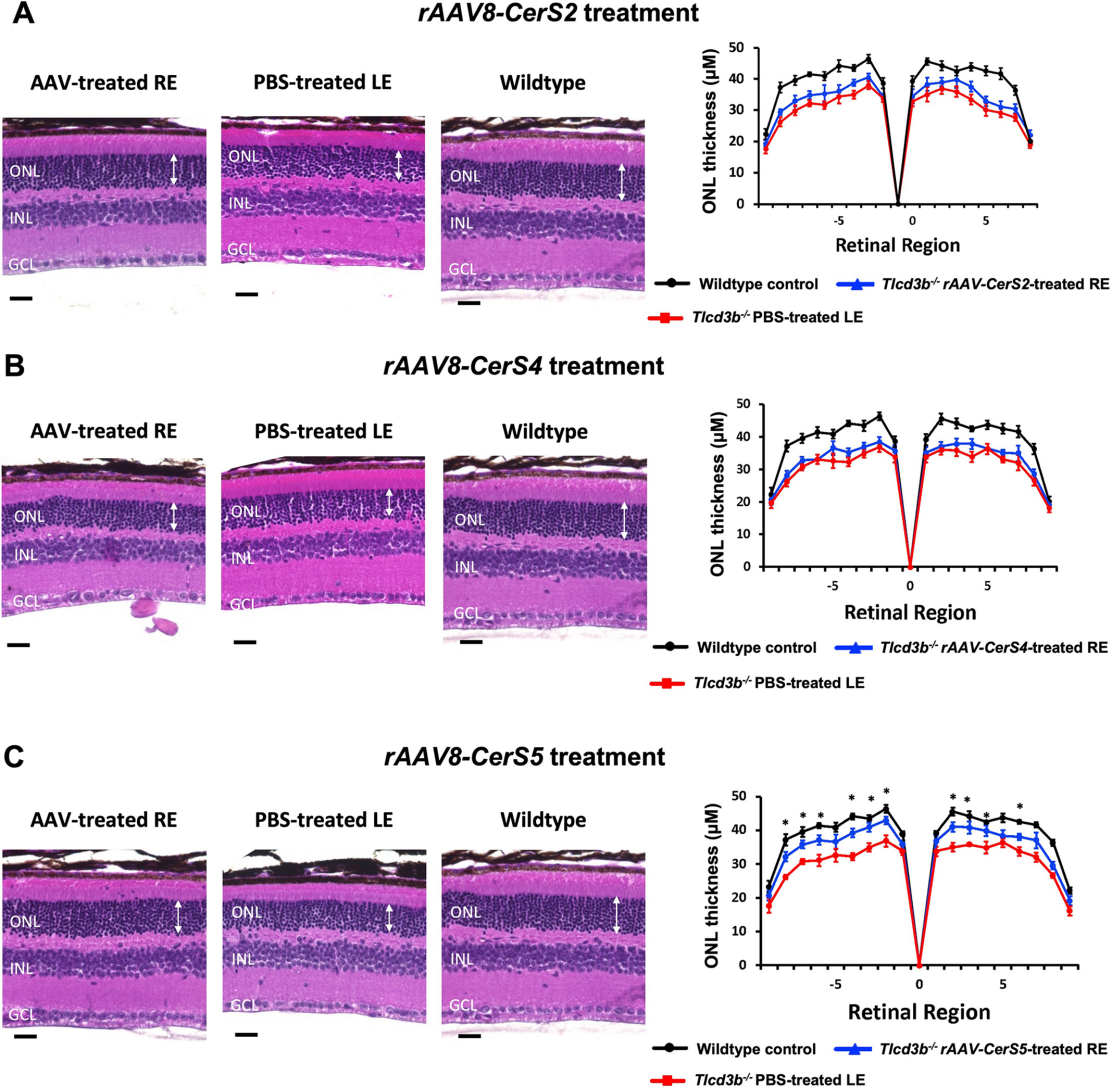
***rAAV8-CerS2* delivery significantly restored the outer nuclear layer thickness in 7-month-old *Tlcd3b^−/−^* mice.** (A-C) For *rAAV8-CerS2*-treated mice (A), *rAAV8-CerS4*-treated mice (B) and *rAAV8-CerS5*-treated mice (C), H&E staining on paraffin-embedded retinal sections was performed to assess morphologic changes in the PBS-treated LEs and CerS-overexpressing REs of 7-month-old *Tlcd3b^−/−^* mice. The same set of wild-type control mice was used for A-C, with the same representative H&E staining image shown for this control in each panel. Scale bars: 50 µm. GCL, ganglion cell layer; INL, inner nuclear layer; ONL, outer nuclear layer. ONL thickness (marked by white arrows) was measured, and the quantification results were plotted using butterfly plots. Only the rescue dosage data of the three treatment groups were plotted in this figure. The data including the other two tested dosages for each group can be found in Fig. S3. The butterfly plots include ONL thickness measured from 18 equally spaced positions along the vertical median of the retina. Each dot represents an individual data point plotted over mean±s.e.m. Position 0 corresponds to the optic nerve head. Error bars denote the s.e.m. For all CerS treatment groups, the ONL thickness of PBS-injected LEs was significantly different from that of wild-type controls. For *rAAV8-CerS2*-treated and *rAAV8-CerS4*-treated mice, no measured data point showed significant difference from the PBS-treated controls. Two-tailed unpaired Student’s *t*-tests were performed at each point. For *rAAV8-CerS5*-treated mice, *P<0.05 for PBS-injected LEs versus AAV-treated REs. A two-way repeated measures ANOVA with Sidak post hoc test for multiple comparisons was also performed (*P*=0.007). *n*=6 mice were used to assay LEs and REs. *n*=6 wild-type mice were used as technical controls.

To determine whether the *rAAV8-CerS* treatments could restore retinal morphology in *Tlcd3b^−/−^* mice, histological analyses on Hematoxylin and Eosin (H&E)-stained retinal sections of both the LEs and REs of *Tlcd3b^−/−^* mice were performed at 7 months of age. As expected, untreated *Tlcd3b^−/−^* LEs showed a reduction in overall retinal thickness as well as in the ONL thickness compared to those of the wild-type control (Fig. 3). For the AAV-treated REs, only the medium-dosage *rAAV8-CerS5* treatment (7.5×10^11^ gc/ml; later referred as ‘rescue dosage’) successfully preserved the retinal and ONL thickness of REs compared to that of the LE contralateral control (Fig. 3C). Upon the treatment of *rAAV8-CerS5* at the medium dosage, *Tlcd3b^−/−^* REs showed significantly thicker ONLs and an increased number of photoreceptor cell nuclei throughout the retina relative to those of the untreated LE. Overall, *rAAV8-CerS5* treatment restored the ONL thickness to approximately 90% of that of the wild-type retina based on the quantitative morphometric analysis on retinal cross-sections.

*rAAV8-CerS2* and *rAAV8-CerS4* treatments, however, failed to rescue retinal morphology (Fig. 3A,B) even at their functional rescue dosages (1.38×10^11^ gc/ml and 1.03×10^10^ gc/ml, respectively, as shown in Fig. S3). For all CerS treatment groups, the high dosage (later referred to as ‘toxic dosage’) failed to rescue and instead drastically reduced the retinal thickness as well as ONL thickness, suggesting toxicity of *rAAV8-CerS* overexpression (Fig. S3).

### Retention of cone and synaptic ribbon morphologies in the retinas of *Tlcd3b^−/−^* mice

**Fig. 4.**
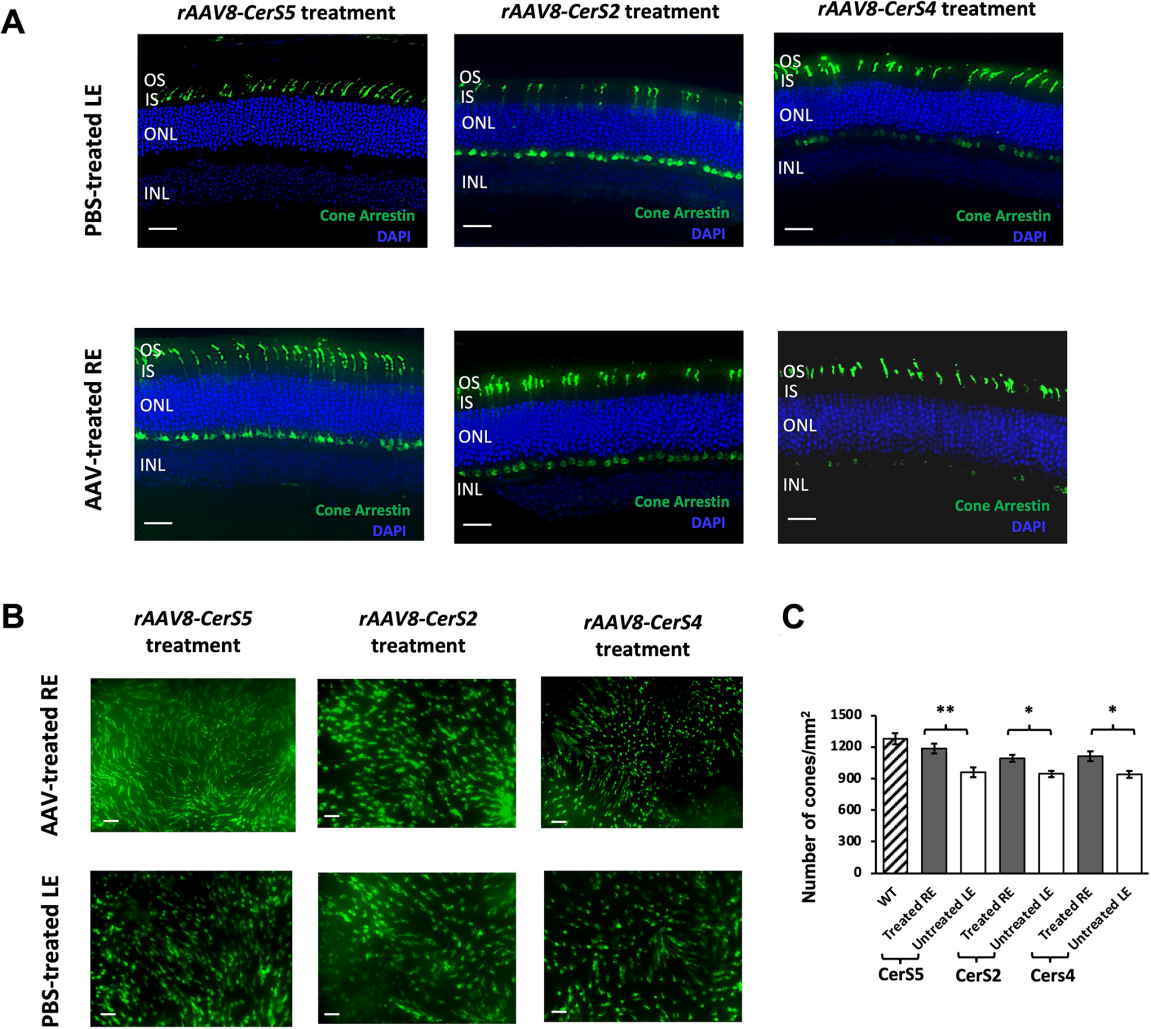
**Cone cells were best preserved by *rAAV8-CerS5* treatment in 7-month-old *Tlcd3b ^−/−^* mice.** (A) Immunofluorescence of cone arrestin (green) was performed on cross-sections of representative contralateral PBS-treated LEs and AAV-treated REs of *Tlcd3b^−/−^* mouse retinas at 7 months of age (*n*=4 for AAV-treated RE and *n*=4 for PBS-treated LE). Scale bars: 20 µm. (B) PNA staining (green) was performed on the whole mounts of retinas treated with different CerS-expressing vectors. Scale bars: 20 µm. (C) Cones were counted in the dorsonasal, dorsotemporal, ventronasal and ventrotemporal regions of PNA-stained (green) retinal whole mounts of AAV-treated REs (*n*=4), PBS-treated LE (*n*=4) and age-matched wild-type (WT) controls (*n*=4), and then normalized as the average number of cones per mm^2^ (mean±s.e.m.). A two-way repeated measures ANOVA with Sidak post hoc test for multiple comparisons was performed between AAV-treated REs and PBS-treated LEs of *Tlcd3b^−/−^* mice for each region (dorsonasal, dorsotemporal, ventronasal and ventrotemporal) to determine statistical significance (*P*=0.004 for *rAAV8-CerS5*; *P*=0.01 for *rAAV8-CerS2*; *P*=0.02 for *rAAV8-CerS4*). *P<0.05; **P<0.01. INL, inner nuclear layer; IS, inner segment; ONL, outer nuclear layer; OS, outer segment.

To assess whether *rAAV8-CerS* treatments successfully retain cone photoreceptor cell integrity and slow down the progression of cone cell degeneration in *Tlcd3b^−/−^* mice at their rescue dosages, cone-arrestin staining on cross-sections and peanut agglutinin (PNA) staining on retinal whole mounts were conducted to examine cone photoreceptor cell morphology. Cone cells were best preserved by *rAAV8-CerS5* treatment, as indicated by the significantly increased number of cones by 30% in REs compared to that in LEs (Fig. 4B,C). Additionally, cones in RE also showed longer outer segments as well as stronger staining in the cone synaptic terminals (Fig. 4A; Fig. S4).

For *rAAV8-CerS2*- and *rAAV8-CerS4*-treated retinas, there was also an increase in the number of cone cells in REs compared to that in LE (22% increase for *rAAV8-CerS2* and 20% increase for *rAAV8-CerS4*), but the increase was less significant compared to what we observed in *rAAV8-CerS5*-treated REs (Fig. 4B,C). Although cone cell numbers were significantly improved, cone morphology was not as successfully retained in REs compared to those that received *rAAV8-CerS5* treatments. As shown in Fig. 4A and Fig. S4, although cone synaptic terminals were well preserved in REs, the outer segments were much shorter and displayed weaker staining relative to those observed in *rAAV8-CerS5*-treated REs.

**Fig. 5.**
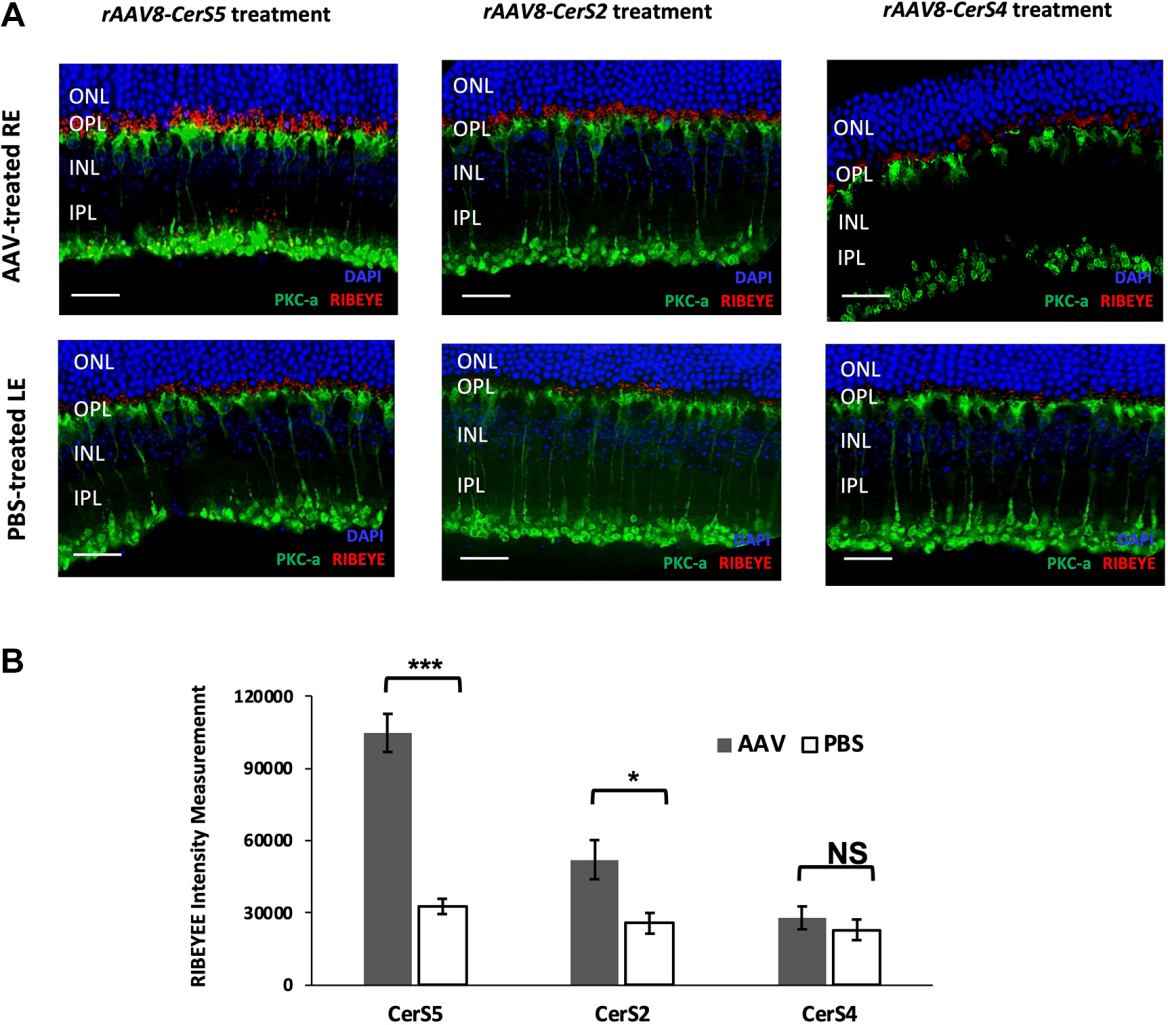
**Bipolar cell and synaptic ribbon morphology in the retinas of 7-month-old *Tlcd3b^−/−^* mice for different CerS overexpressions.** (A) Immunofluorescence against PKC-a and RIBEYE was performed to visualize rod bipolar cells and synaptic ribbons. A stronger synaptic ribbon staining in the *rAAV8-CerS5*-treated eye indicated improved phototransduction, which was consistent with the enhanced scotopic b-wave amplitudes of ERG results. The synaptic ribbons, identified with anti-RIBEYE (red), are located on top of rod bipolar cell tip terminals (identified with PKC-α staining in green). INL, inner nuclear layer; IPL, inner plexiform layer; ONL, outer nuclear layer; OPL, outer plexiform layer. Scale bars: 20 μm. (B) RIBEYE intensity was measured from six equally spaced regions along the vertical median of the retina (*n*=6 for AAV-treated REs and *n*=6 for PBS-treated LEs) and quantified using ImageJ. Error bars denote the s.e.m. Two-tailed unpaired Student’s *t*-tests were performed. NS, not significant; *P<0.05; ***P<0.001.

Given that *Tlcd3b^−/−^* mice showed scotopic b-wave reductions at 7 months of age, PKC-a (encoded by *Prkca*) and RIBEYE (encoded by *Ctbp2*) co-staining was performed to assess rod bipolar cell morphology and retention of bipolar ribbon synapses. All *rAAV8-CerS* therapy treatment groups led to better preservation of synapses at the rescue dosages, as evident by the more robust RIBEYE staining in AAV-treated REs compared to that in the contralateral control eye (Fig. 5). Among the three *rAAV8-CerS*s that were tested, *rAAV8-CerS5* showed the best preservation of ribbon synapses compared to *rAAV8-CerS2* and *rAAV8-CerS4*, as indicated by the strongest RIBEYE staining.

### The recovery of different retinal ceramide species by *rAAV8-CerS* treatments

To further study the impact of *rAAV8-CerS* treatments on retinal ceramide profiles, we carried out targeted metabolomics analyses to assess the change in levels of different ceramide subtypes and sphingosine in *Tlcd3b^−/−^* mice. For each virus, we looked at two dosages: the rescue dosage and the toxic dosage.

**Fig. 6.**
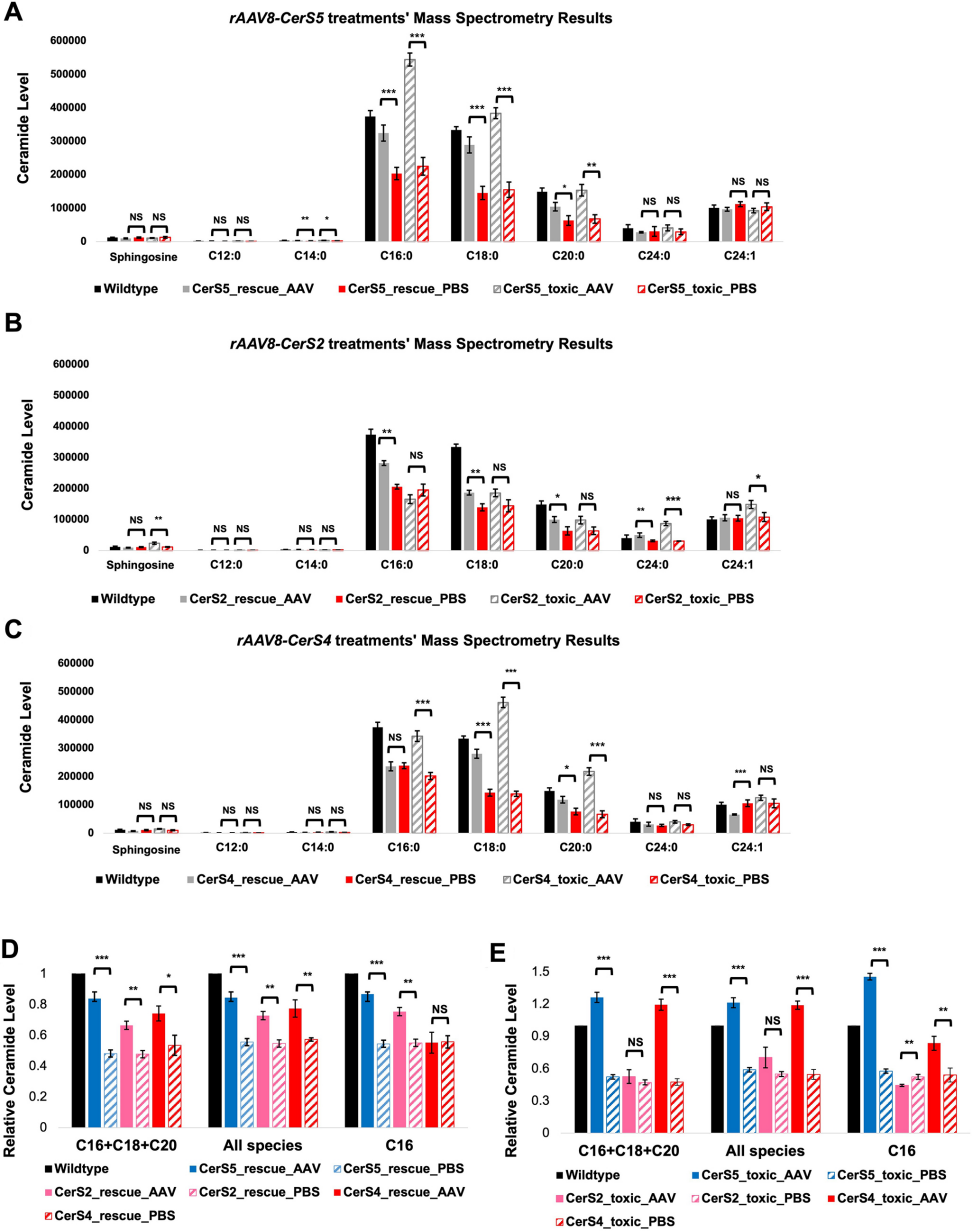
**Mass spectrometric analyses of ceramide species and sphingosine in the retinas of *Tlcd3b^−/−^* mice that were treated with *rAAV8-CerSs* at the rescue and toxic dosages.** For each CerS, two dosages were tested, the ‘rescue dosage’ and the ‘toxic dosage’. In each dosage group, each mouse was injected with AAV in the RE and PBS in the LE. (A-C) Ceramide profiles of *rAAV8-CerS2*-treated mice (A), *rAAV8-CerS4*-treated mice (B) and *rAAV8-CerS5*-treated mice (C). (D,E) The cross-treatment comparisons at the rescue dosages (D) and toxic dosages (E). The *y*-axis shows the relative values of ceramide levels in each category compared to wild-type levels. All retinas were collected when the P21-injected mice were 3 months of age. *n*=5 mice were used to assay LEs and REs. *n*=5 wild-type mice were used as technical controls. The level of sphingolipids was normalized to the total protein content in each sample. All values are mean±s.e.m, and significance was determined by two-tailed unpaired Student's *t*-test. NS, not significant, *P*>0.05; *P<0.05; **P<0.005; ***P<0.001.

CerS5 is reported to generate primarily C16:0 and a minimal amount of C14:0. At the rescue dosage, *rAAV8-CerS5* successfully restored the levels of C14:0, C16:0 and C18:0 ceramides in REs to approximately 85-90% of wild-type levels and recovered C20:0 ceramides to approximately 70% of wild-type levels (Fig. 6A). Nevertheless, overexpressing CerS5 at the rescue dosage did not induce the overproduction of other ceramide species. Both observations gave supportive evidence backing up the best rescue effects of *rAAV8-CerS5*. At the toxic dosage, *rAAV8-CerS5* triggered the overproduction of all three major ceramide species in the retina, C16:0, C18:0 and C20:0, which led to the toxicity and retinal degeneration of the REs.

The rescue dosage of *rAAV8-CerS2*, however, triggered an increase in the levels of C24:0 (Fig. 6B), which is expected as it belongs to the ceramide profile of CerS2. It also elevated the levels of C16:0, C18:0 and C20:0 in the RE compared to the LE. At the toxic dosage, *rAAV8-CerS2* triggered the overproduction of its profile ceramide, C24:0 (more than two times the levels seen in wild type), as well as C24:1 (approximately 1.5 times the levels in wild type) and sphingosine, but did not cause further increase in the levels of C16:0, C18:0 and C20:0 compared to what was observed at the rescue dosage. Interestingly, C16:0 levels decreased to levels even lower than those of the contralateral left eye.

CerS4 synthesizes C18:0-C22:0 ceramides. On the one hand, as expected, the rescue dosage triggered an increase in the level of C18:0 and C20:0 in the treated RE compared to the PBS-injected LE, to approximately 80% of the wild-type levels. On the other hand, it significantly decreased the level of C24:1 (Fig. 6C). However, at the toxic dosage, *rAAV8-CerS4* triggered a somewhat global change in ceramide levels. There was an overproduction of all ceramides relative to their levels in the wild-type control, except for C12:0, C24:0 and C24:1. Specifically, the levels of its profile ceramides, C18:0 and C20:0, were elevated to roughly 1.4 times the levels in wild-type control.

In terms of total ceramide levels, *rAAV8-CerS5* treatment also achieved the best restoration of all tested ceramide species and the sum of TLCD3B profile ceramides, C16:0, C18:0, and C20:0, to more than 80% of wild-type levels at the rescue dosage (Fig. 6D). However, despite *rAAV8-CerS2* treatment having slightly better functional and morphological retention in the retina, the relative increase in the overall ceramide levels as well as that of TLCD3B profile ceramides were lower than those of *rAAV8-CerS4*-treated retinas (Fig. 6D), suggesting that the changes in the overall ceramide levels are not a major contributor to retinal degeneration in the context of TLCD3B loss of function. Interestingly, the C16 ceramide levels of *rAAV8-CerS4*-treated retinas remained unchanged compared to its levels in PBS-treated retinas, but *rAAV8-CerS2*-treated retinas demonstrated much higher C16 ceramide levels. At the toxic dosages, *rAAV8-CerS5* treatment caused the highest level of ceramide overproduction (approximately 23% higher for overall ceramide levels and 27% higher for TLCD3B profile ceramide levels compared to those of wild type), followed by *rAAV8-CerS4* treatment, which was approximately 20% higher for overall ceramide levels and 19% higher for TLCD3B profile ceramide level compared to those of wild type (Fig. 6E). Interestingly, although the toxic dosage of *rAAV8-CerS2* did not drive further changes in overall ceramide levels, it led to a 10% decrease in C16 ceramide levels compared to its levels in rescue dosage-treated REs (Fig. 6E).

## DISCUSSION

We report that the compensation of ceramides through the delivery of different *rAAV8-CerS*s successfully alleviated both impaired retinal function and photoreceptor degeneration of *Tlcd3b^−/−^* mice. Specifically, *rAAV8-CerS5* had the best rescue potential, given that it demonstrated the best ERG response restoration, ONL thickness retention and preservation of cone photoreceptor cells as well as synaptic ribbons. In addition, the targeted metabolomics analyses showed that at the rescue dosage, *rAAV8-CerS5* best recovered both the TLCD3B profile ceramides and overall ceramide levels, which can be explained by CerS5 having the largest profile overlap with TLCD3B relative to that of CerS2 and CerS4.

Previous studies have mainly focused on the link between overall ceramide level and activation of apoptosis in photoreceptors. However, there is a gap in the understanding of roles each ceramide subtype plays. It is known that different CerSs are highly interactive with each other, and the overexpression of one CerS may enhance the ceramide subtypes produced by the other CerSs (Zhao et al., 2011). Interestingly, all three overexpressed CerSs induced an increase in the levels of ceramide subtypes that were not under their respective profiles. For CerS4 and CerS5, their impact appeared to be on subtypes with a similar chain length. CerS2 overexpression, however, had a global impact both on ceramides with different chain lengths and on sphingosine, which is the backbone and the intermediate between ceramide and sphingosine-1-phosphate conversion, suggesting that CerS2 may have a more universal role in ceramide synthesis relative to the roles of CerS4 and CerS5.

Our previous *rAAV8-Tlcd3b* gene therapy study suggested a key role played by ceramides and the association between TLCD3B regulation and ceramide profile change (Qian et al., 2022). In this study, we further demonstrated that in the context of *Tlcd3b*-associated retinal degeneration, the disruption of overall ceramide level is not the leading cause of photoreceptor cell death. Instead, different ceramide species may play distinct roles and the balance among them may be the key to retinal health in *Tlcd3b^−/−^* mice. At the rescue dosages, *rAAV8-CerS2* restored overall ceramide levels and TLCD3B profile ceramides to a lesser extent compared to *rAAV8-CerS4* treatment, yet *rAAV8-CerS2* treatment had better functional rescue and significantly improved cone photoreceptor cell preservation. There are three possible explanations for the improved rescue potential of *rAAV8-CerS2*. First, *rAAV8-CerS4* treatment failed to restore C16:0, which is the major TLCD3B profile ceramide and the most dominant ceramide subtype in the retina. Given the abundance of C16:0 ceramide in the normal retina and the severe C16:0 reduction owing to TLCD3B loss of function in *Tlcd3b^−/−^* mice, C16:0 ceramide might be the leading player causing retinal degeneration, and a higher compensation by C18:0 and C20:0 ceramides does not rescue retinal degeneration as effectively as C16:0 ceramide restoration. Another possibility is that when increasing the level of different ceramide species, these species need to be kept at a certain ratio to maintain homeostasis. Although *rAAV8-CerS5*-treated REs clearly demonstrated a descending level of the C16:0, C18:0 and C20:0 ceramides detected and *rAAV8-CerS2*-treated REs followed a similar pattern, *rAAV8-CerS4*-treated REs broke the pattern by failing to elevate C16:0 production. A third possibility would be that ceramides with different chain lengths may have different roles in terms of activating or preventing photoreceptor cell apoptosis. Some studies reported that ceramides with longer chain lengths (>C20) may have a beneficial impact on cell survival (Mesicek et al., 2010; Hartmann et al., 2012). It is possible that an elevated level of C24:0 in *rAAV8-CerS2*-treated REs mitigated the detrimental impact caused by the loss of TLCD3B profile ceramides. Collectively, all these possibilities point to the delicacy of retinal ceramide subtype homeostasis in the context of *Tlcd3b^−/−^* mice, highlighting the importance to look not only at the overall ceramide level changes, but also at ceramides at the subtype level for retinopathies. As fluctuations of each ceramide subtype may have a complicated impact on the ceramide metabolic ecosystem in the retina, this further implicates the complexity of the sphingolipid pathways. Therefore, the exact relationships between different ceramide subtypes and their exact role in retinal degeneration still remains unanswered and requires further study.

In conclusion, our results showed that the subretinal injections of all three *rAAV8-CerS*s successfully restored retinal functions, but only *rAAV8-CerS5* was able to preserve retinal morphology, indicating that CerSs and TLCD3B play partially redundant roles (Qian et al., 2022). However, *Tlcd3b^−/−^* mice demonstrated a greater sensitivity to dosage variations in the case of *rAAV8-Tlcd3b* treatments than *rAAV8-CerSs* treatments. Additionally, the sensitivity of different phenotypes of *Tlcd3b^−/−^* mice also varied under treatments by the three *rAAV8-CerS*s tested, suggesting the delicacy of regulating and maintaining the level of each ceramide species. Findings from this research suggested that ceramide is an effective target for molecular therapy development for patients who have retinal pathologies and for those with *TLCD3B* mutations. Future studies will explore more direct ways of ceramide delivery into the retina and test the best combination and dosage of different ceramide subtypes for treatments on *Tlcd3b*-associated retinopathies.

## MATERIALS AND METHODS

### Production of *rAAV8-CerS* viral vectors

cDNAs encoding full-length CerS2, CerS4 and CerS5 (GenScript OMu18716, OMu21307 and OMu18680) were sequence verified and amplified by PCR. For detection of exogenous CerS transgene expression, a sequence encoding an N-terminus FLAG tag was present in the original GenScript vectors, and PCR amplification was performed using primer sets that included the sequences of the FLAG tag and the AgeI and EcoRI restriction enzyme sites at the ends (Fig. S1A). Both the pTR-hGRK1 AAV vector (Addgene, plasmid #60957; see vector map in Fig. S1B) and the PCR-amplified cDNA were digested with AgeI and EcoRI. The digested vector was gel-purified and then ligated with the digested insert. For AAV packaging, rAAV8 (Gene Vector Core, Baylor College of Medicine) was used for packaging *hGrk1-CerS* genes to achieve robust transduction efficiency and expression in retinal photoreceptors (Gene Vector Core, Baylor College of Medicine).

### Animals

*Tlcd3b^−/−^* mice were generated as previously described (Bertrand et al., 2021) and maintained on a C57BL/6J genetic background. All mice in this study were maintained in a 14-h light/10-h dark cyclic environment. All animal operations were approved by the Institutional Animal Care and Use Committee at Baylor College of Medicine. For all treatment groups, both sexes of mice were examined.

### Subretinal injection

P21 mice were anesthetized with Rodent III (Center for Comparative Medicine, Baylor College of Medicine), a combination drug consisting of ketamine (22 mg/kg), xylazine (4.4 mg/kg) and acepromazine (0.37 mg/kg), which was injected intraperitoneally. Subretinal injections were performed as described previously (Zhong et al., 2015). A shallow incision was made through the sclera with a beveled 30-gauge needle. A 32-gauge blunt needle was presented inside the vitreous cavity and pushed forward until the tip of the needle had moved past the retina. The viral solution was injected into the subretinal space using an Ultra-Micro-Pump II and Micron-4 Controller (World Precision Instruments, Sarasota, FL, USA). All mice were treated once in the subretinal space with 1 µl *rAAV8-hGRK1-CerS* vectors (*rAAV8-CerS2*, *rAAV8-hGRK1-CerS4* or *rAAV8-hGRK1-CerS*) in the RE, whereas the contralateral LE served as an internal control and was injected subretinally with 1 µl PBS. Age-matched wild-type mice were used as the external controls.

### qPCR analysis of CerS mRNAs

Total RNA used in the study was extracted from the retinal tissues of 7-month-old mice using the TRIzol reagent (Invitrogen, 15596018). For each *rAAV8-CerS* treatment, three mice were used (three AAV-treated REs and three PBS-treated LEs). cDNA synthesis was performed with 1 µg of total RNA according to the manufacturer's protocol (Invitrogen, 18091050). *Gapdh* (forward primer, 5′-GAGTGGGAGTTGCTGTTGAAGT-3′, and reverse primer, 5′-AGGATAGTCATTTTGGGGTTTGT-3′) was used as an internal control in SYBR Green PCR Master Mix kits (Thermo Fisher Scientific, 4367659). The relative RNA levels of *Cers2*, *Cers4* and *Cers5* in AAV-treated eyes (test groups) and PBS-treated eyes (control groups) were determined using the CFX Opus 96 Real-Time PCR System (Bio-Sad, #12016658) and the following primers: *Cers2* (forward, 5′-GGACCGGTGCCACCATGCTCCAGACCTTGTATGA-3′, and reverse, 5′-GGGGAATTCTCAGTCATTCTTAGGATGATT-3′), *Cers4* (forward, 5′-GGACCGGTGCCACCATGTCGTTCAGCTTGAGTGAG-3′, and reverse, 5′-GGGAATTCTGTCTATGTGGCCCGGGTGT-3′) and *Cers5* (forward, 5′-GGACCGGTGCCACCATGGCGACTGCAGCAGCG-3′, and reverse, 5′-GGGAATTCCTAGTCACAGGAGTGTAGAT-3′). All experiments were carried out in triplicates. Ct values were extracted using the Bio-Rad CFX Maestro Software to calculate the relative quantitative values, which were shown by 2^−ΔΔCt^. Two-tailed unpaired Student’s *t*-tests were performed for statistical analyses.

### Electroretinography analysis

Mice were dark adapted overnight before ERG acquisition, and all procedures were performed under dim red light. Mice were anesthetized and topical tropicamide (1.0%; Generic, 1168129) and phenylephrine hydrochloride (2.5%; Bausch & Lomb Americas, 42702010210) eye drops were used to dilate the pupils. The cornea was anesthetized with proparacaine hydrochloride (0.5%) eye drops (Generic, 2963726). Goniosoft (2.5%; McKesson, 1122510) was applied to the cornea to keep the eyes hydrated. A ground electrode was placed on mice subcutaneously. ERGs were recorded using electrodes (LKC Technologies, Gaithersburg, MD, USA; 95-033 M) placed at the center of each cornea. Data acquisition and storage were conducted using the LKC UTAS Visual Diagnostic System and EMWIN software (LKC Technologies). The oscillatory potential was isolated and removed using the EMWIN software prior to wave analysis. A two-way repeated measures ANOVA with Sidak post hoc test for multiple comparisons was performed between AAV-treated REs and PBS-treated LEs of *Tlcd3b^−/−^* mice to determine group-to-group statistical significance, whereas two-tailed unpaired Student’s *t*-tests were conducted for each luminescence to determine statistical significance.

### Immunohistochemical analysis

Mice were euthanized with isoflurane, followed by cervical dislocation. Eyes were enucleated and fixed in fresh Davidson's fixative [(20% formaldehyde (Sigma-Aldrich, 252549), 35% ethanol, 10% acetic acid (Sigma-Aldrich, 695092), and 53% ddH_2_O)] overnight at 4°C. After fixation, eyes were dehydrated in ethanol series and embedded in paraffin wax according to standard protocol (Qian et al., 2022). The paraffin-embedded tissue was then cut into 7-μm-thick sections (Leica, RM2255).

H&E staining was performed on retinal cross-sections, and stained sections were visualized using light microscopy (Zeiss Apotome). ONL thickness was measured using the Zen software (Zeiss). A two-way repeated measures ANOVA with Sidak post hoc test for multiple comparisons was performed between AAV-treated REs and PBS-treated LEs of *Tlcd3b^−/−^* mice to determine group-to-group statistical significance, whereas two-tailed unpaired Student’s *t*-tests were conducted for each position to determine statistical significance.

For immunofluorescence staining, paraffin-embedded retinal sections were prepared as described above. Following deparaffinization and rehydration, antigen retrieval was performed by boiling retinal sections in citrate buffer [for 1l citrate buffer, it is composed of 800 ml ddH_2_O, 24.268g sodium citrate dihydrate (Sigma-Aldrich, 567446), 3.358g citric acid (Sigma-Aldrich, 791725), adjusting pH to 7.4 using 0.1 N hydrochloric acid] for 30 min. Retinal sections were washed in PBS five times for 5 min each. Retinal sections were incubated in hybridization buffer NGST (10% normal goat serum with 0.1% Triton-X 100 in PBS) for 1 h at room temperature. Next, sections were incubated in primary antibodies (anti-FLAG antibody, Sigma-Aldrich, F3165, 1:500; anti-cone arrestin, Sigma-Aldrich, AB15282, 1:500; Anti-Pkca, Sigma-Aldrich, P4334, 1:5000; or anti-RIBEYE, BD Biosciences, 612044,1:1000) or Alexa Fluor 488-conjugated PNA (Invitrogen, L21409, 1:400) diluted in NGST overnight at 4°C. The following steps were conducted in the dark. Retinal sections were incubated in secondary antibodies [anti-Cy5-rabbit (Invitrogen, A21242) or anti-Cy3-mouse (Invitrogen, A32728)] diluted to 1:400 in NGST for 2 h at room temperature and then washed in PBS five times for 5 min each. All sections were then counterstained with DAPI diluted 1:1000 in PBS for 15 min at room temperature and then washed in PBS four times for 2 min each. Sections were air dried for 5 min at room temperature before ProLong Gold mounting medium (Life Technologies, P36934) was applied to each section, and slides were coverslipped. Stained sections were visualized using fluorescence microscopy (Zeiss Axio Observer Z1).

For retinal whole-mount staining, eyes were then fixed in 4% formaldehyde in PBS for 1 h at 4°C. Following fixation, retinal cups were dissected and incubated in NGST overnight at 4°C. The following steps took place in the dark. Retinal cups were incubated in Alexa Fluor 488-conjugated PNA (Invitrogen, L21409, 1:400) in NGST for 48 h at 4°C. Retinal cups were washed in PBS three times for 5 min each. Retinal cups were then transferred to a slide, cut into four petals, and flattened with the scleral side up. Samples were visualized using fluorescence microscopy (Zeiss Axio Observer.Z1). A two-way repeated measures ANOVA with Sidak post hoc test for multiple comparisons was performed between AAV-treated REs and PBS-treated LEs of *Tlcd3b^−/−^* mice for each region (dorsonasal, dorsotemporal, ventronasal and ventrotemporal) to determine statistical significance.

### Targeted metabolomics analyses

Mouse retinas were collected at 3 months of age. Simultaneously, retinas of age-matched wild-type mice were collected as the control. For tissue preparation, each retina sample (∼20 mg) was mixed with 200 µl 10× diluted PBS (4°C) and 80 µl internal standard solution [phosphatidylcholine (17:0/17:0) and phosphatidylglycerol (17:0/17:0) in methanol; 50 µM; 4°C] in an Eppendorf tube (1.5 ml). Homogenization (2 min) was performed using a Bullet Blender homogenizer (Next Advance, Averill Park, NY, USA). After homogenization, 400 µl methyl tert-butyl ether (MTBE; Sigma-Aldrich) was added to the sample. Following this, the sample was vortexed for 30 s, stored under −20°C for 30 min, and sonicated in an ice bath for 10 min. After centrifugation (1600 ***g***, 10 min), 500 µl of the upper MTBE layer was collected into a new Eppendorf tube. The MTBE layer was then dried in a Vacufuge Plus Evaporator (Eppendorf, 022820168), and samples were reconstituted with 100 μl 1:1 CHCl_3_:methanol. A quality-control sample was prepared by pooling all study samples, and it was measured every ten samples to ensure consistent output by a liquid chromatography tandem mass spectrometry (LC-MS/MS) system. All mass spectrometry experiments were done on an Agilent 1290 LC-6490 QQQ-MS (Santa Clara, CA, USA). Standard sphingolipid compounds were purchased from Thermo Fisher Scientific, Sigma-Aldrich and Avanti Polar Lipids (Alabaster, AL, USA) in order to confirm detected lipids. Reverse-phase chromatography was used with a Waters XSelect HSS T3 column (150×2.1 mm, 2.5 µm particle size; Waters Corporation, Milford, MA, USA). The flow was maintained at 0.3 ml/min. The mobile phase solvent A consisted of 10 mM ammonium acetate in 60% H_2_O/40% acetonitrile (ACN). Solvent B consisted of 10 mM ammonium acetate in 90% isopropyl alcohol/10% ACN. Isocratic elution was used with 50% solvent B for 3 min, before its percentage was gradually increased to 100% over the next 12 min. Following 10 min of continued 100% solvent B, at t=25 min, the percentage of solvent B was decreased gradually back to 50% to prepare for the next sample injection. MS data were extracted using Agilent's MassHunter Quantitative Analysis for QQQ (Santa Clara, CA, USA). Finally, the levels of sphingolipids were normalized to the protein amount of each retina sample (in micrograms), which was measured using the BCA protein assay kit (Thermo Fisher Scientific, PA198579). Statistical significance was determined by two-tailed unpaired Student's *t*-test for each lipid species.

## Supplementary Material

10.1242/dmm.050168_sup1 Supplementary information

## Data Availability

All relevant data can be found within the article and its supplementary information.
