# Supplementary material for: Ceramide compensation by ceramide synthases preserves retinal function and structure in a retinal dystrophy mouse model
Source: Dis Model Mech. 2023 Jul 19;16(7):dmm050168. doi: 10.1242/dmm.050168 (PMC10387349; doi:10.1242/dmm.050168)
Supplement: Supplementary information [file dmm-16-050168-s1.pdf]

(A)

CerS2 forward primer: GGACCGGTGCCACCATGCTCCAGACCTTGTATGA
CerS2 reverse primer: GGGGAATTCTCACTTATCGTCGTCATCC

CerS4 forward primer: GGACCGGTGCCACCATGTCGTTCAGCTTGAGTGAG
CerS4 reverse primer: GGGGAATTCTCACTTATCGTCGTCATCC

CerS5 forward primer: GGACCGGTGCCACCATGGCGACTGCAGCAGCG
CerS5 reverse primer: GGGGAATTCTCACTTATCGTCGTCATCC

(B)

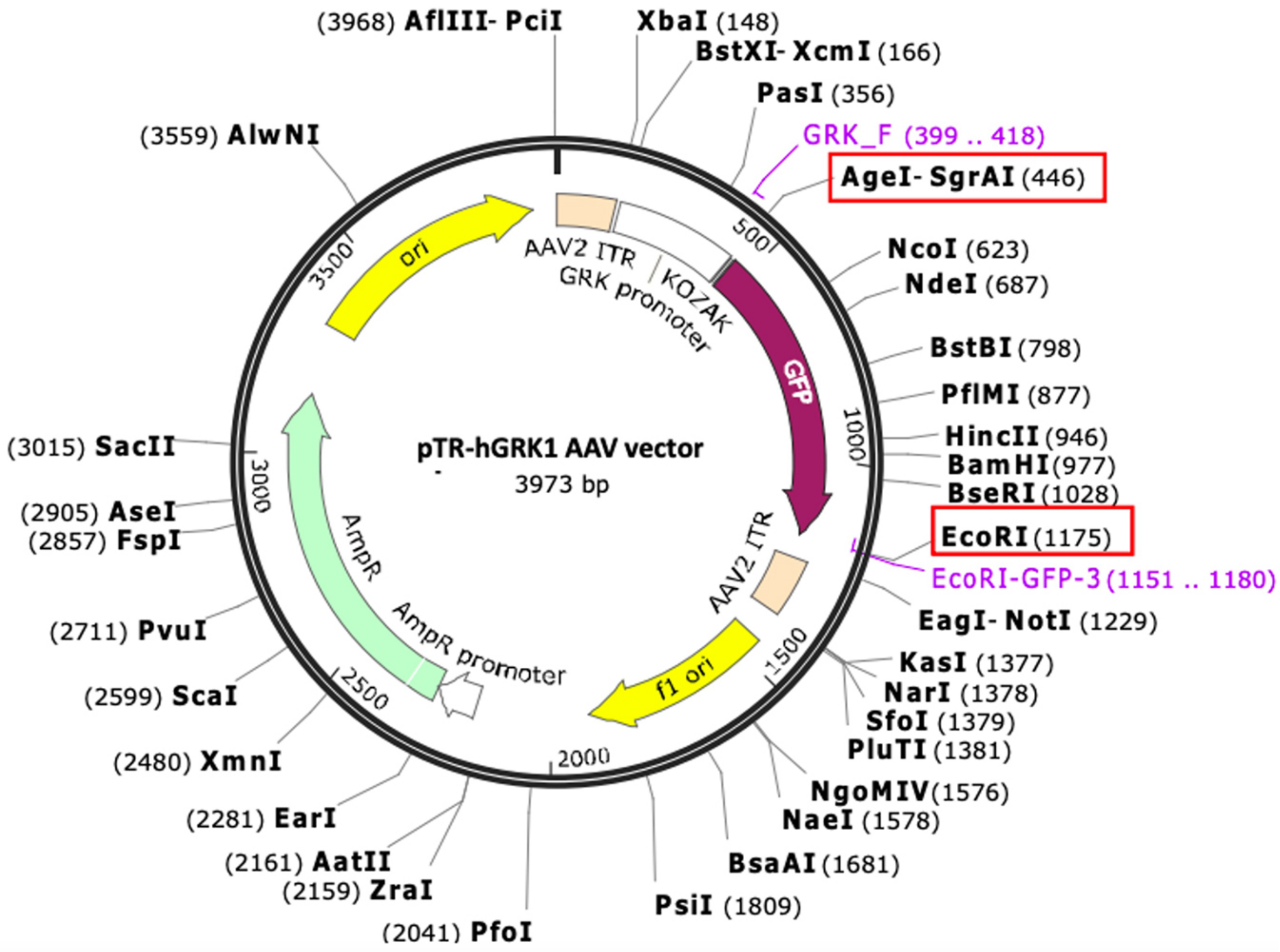

Fig. S1. Cloning primers and vector map. (A) primers that were used for PCR amplification (B) The plasmid map of pTR-hGRK1 AAV vector. The restriction enzyme sites, AgeI and EcoRI, are circled in red. During cloning, the GFP sequence was replaced by the cDNA of CERS2, CERS4, and CERS5.

Disease Models & Mechanisms • Supplementary information

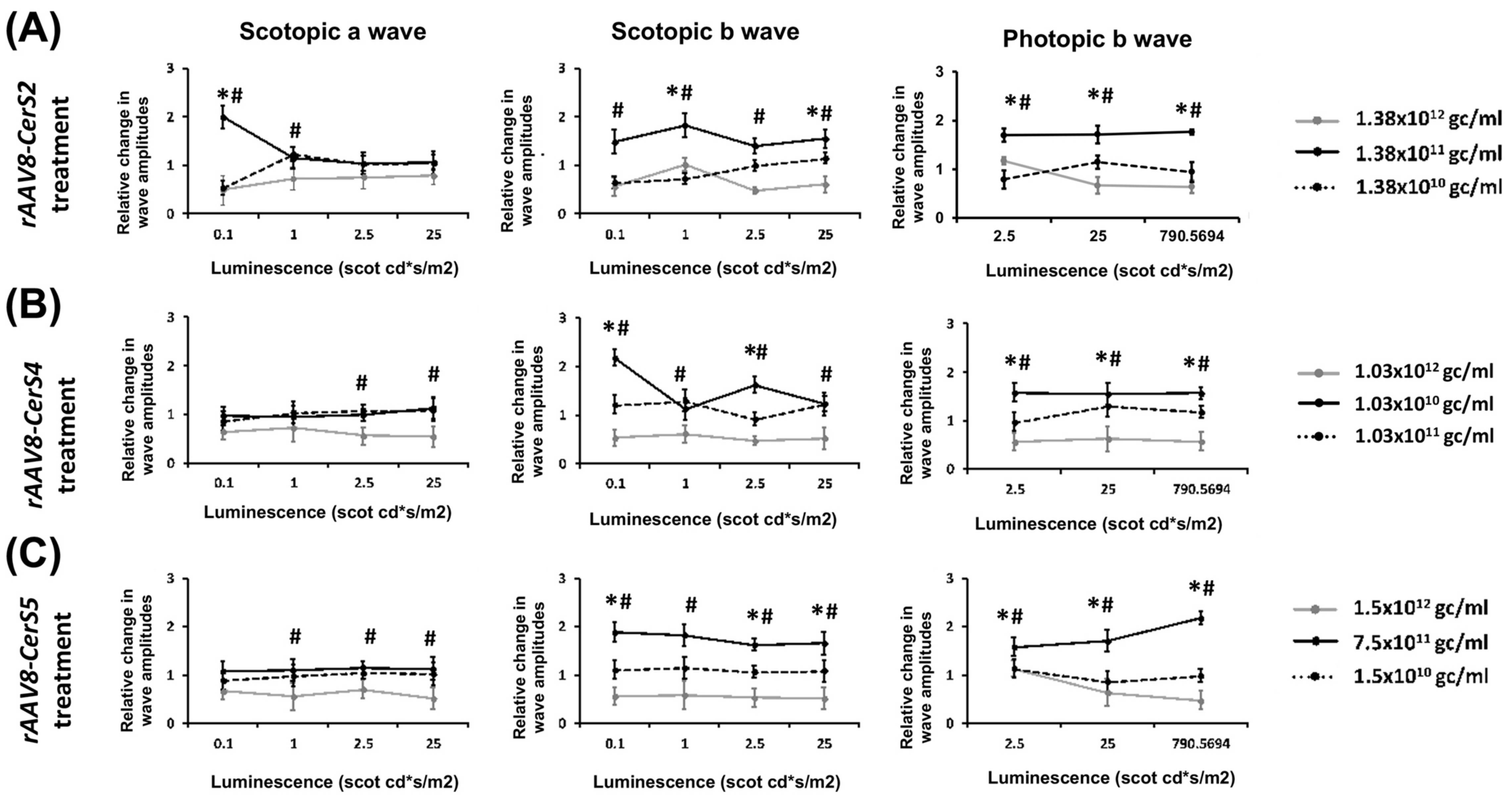

Fig. S2. ***rAAV8-CerS*****s treatments demonstrated dosage-dependent impact on retinal photoresponses in *Tlcd3b$^{-/-}$* mice**. Quantitative evaluation of full-field electroretinography (ERG) were performed on 7-month-old dark-adapted *Tlcd3b$^{-/-}$* mice that were treated by subretinal injections of (A) *rAAV8-CerS2* (B) *rAAV8-CerS4* and (C) *rAAV8-CerS5* at P21. n = 6 mice were used to assay LE and RE ERGs. n = 4 wild-type mice were used as technical controls. The line plots were plotted as the value of the ERG wave amplitudes in AAV-injected RE compared to the PBS-injected LE at each recorded luminescence. A value of 1 means the RE photoresponses is the same as that of LE, indicating no change relative to LE photoresponses. A value smaller than 1 means the treatment has a negative impact on the ERG responses, while a value greater than 1 indicates a positive impact. For *rAAV8-CerS2*, the concentrations tested were: high-dosage group: 1.38×10^{12}gc/ml (toxic); medium dosage group: 1.38×10^{11}gc/ml (rescue); low-dosage group: 1.38×10^{10}gc/ml (no effect). For *rAAV8-CerS4*, the concentrations tested were: high-dosage group: 1.03×10^{12}gc/ml (toxic); medium dosage group: 1.03×10^{11}gc/ml (no effect); low-dosage group: 1.03×10^{10}gc/ml (rescue). For *rAAV8-CerS25*, the concentrations tested were: high-dosage group: 1.5×10^{12}gc/ml (toxic); medium dosage group: 7.5×10^{11}gc/ml (rescue); low-dosage group: 1.5×10^{10}gc/ml (no effect). Statistical analysis was performed by student t-tests. Statistical analysis is denoted with the following symbols: rescue-dosage-treated RE versus LE (*$p < 0.05$), and toxic-dosage-treated RE versus LE (#$p < 0.05$). Error bars denote the SEM.

Disease Models & Mechanisms • Supplementary information

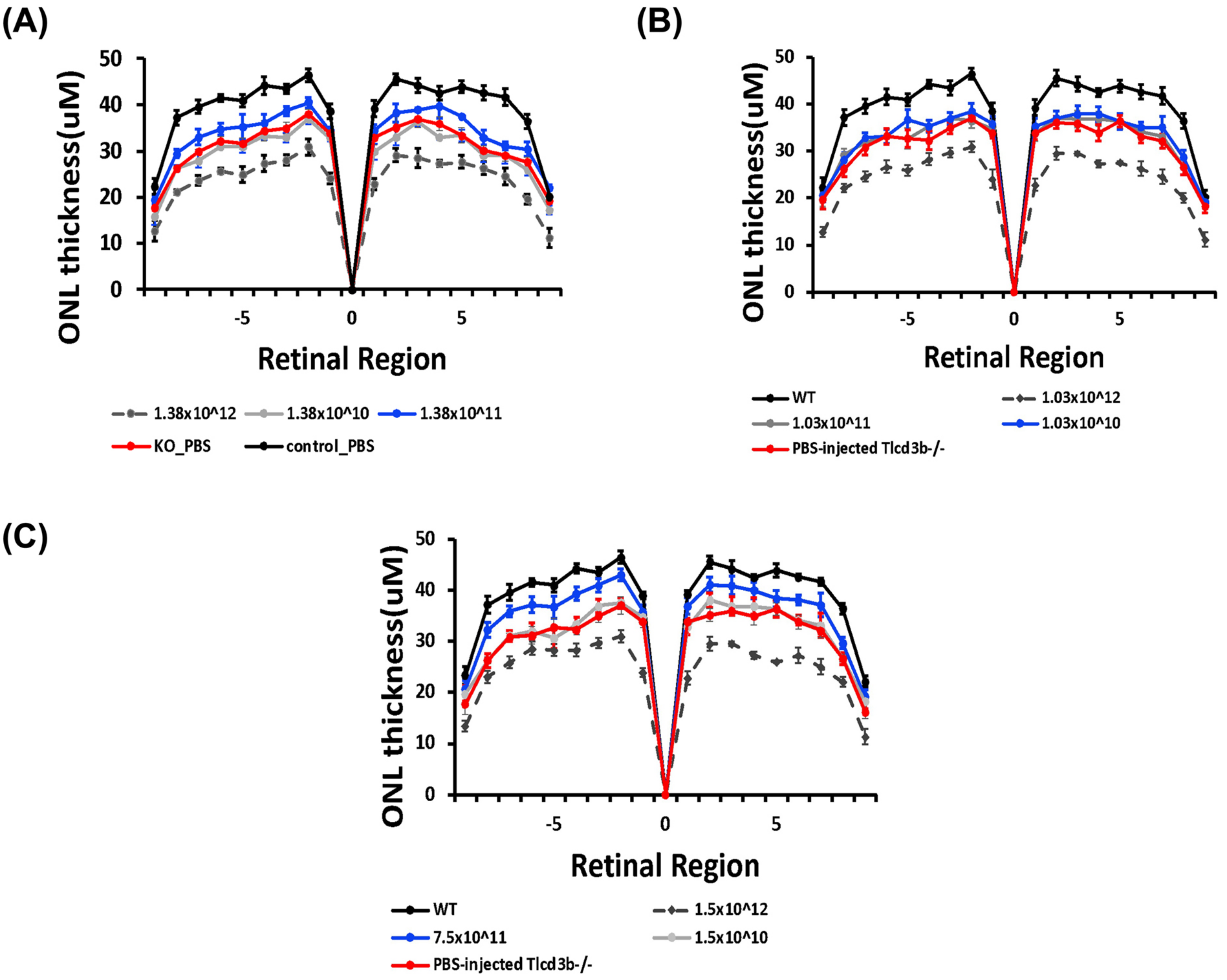

Fig. S3. ONL quantification results of 7M *Tlcd3b$^{-/-}$* mice that were treated with different *rAAV8-CerS*s concentrations. (A) Among the 3 *rAAV8-CerS2* dosages of 1.38 x10^{12} g.c./ml, 1.38 x10^{11} g.c./ml, and 1.38 x10^{10} g.c./ml that were tested, none of them demonstrated restoration of ONL thickness. (B) Similarly, no increase in ONL thickness was observed in all 3 dosage treatment groups of *rAAV8-CerS4* (1.03x10^{12} g.c./ml, 1.03x10^{11} g.c./ml, and 1.03x10^{10} g.c./ml) (C) Among the 3 *rAAV8-CerS5,* dosages of 1.5 x10^{12} g.c./ml, 7.5 x10^{11} g.c./ml, and 1.5 x10^{10} g.c./ml that were tested, only the dosage of 7.5 x10^{11} g.c./ml demonstrated restoration of ONL thickness($p= 0.009$). The ONL quantifications for *rAAV8-CerS2* at a dosage of 1.38 x10^{11} g.c./ml, *rAAV8-CerS4* at a dosage of 1.03x10^{10} g.c./ml, and *rAAV8-CerS5* at a dosage of 7.5 x10^{11} g.c./ml were previously demonstrated in Figure 3 but were plotted here again to provide a better comparison among different dosages tested.

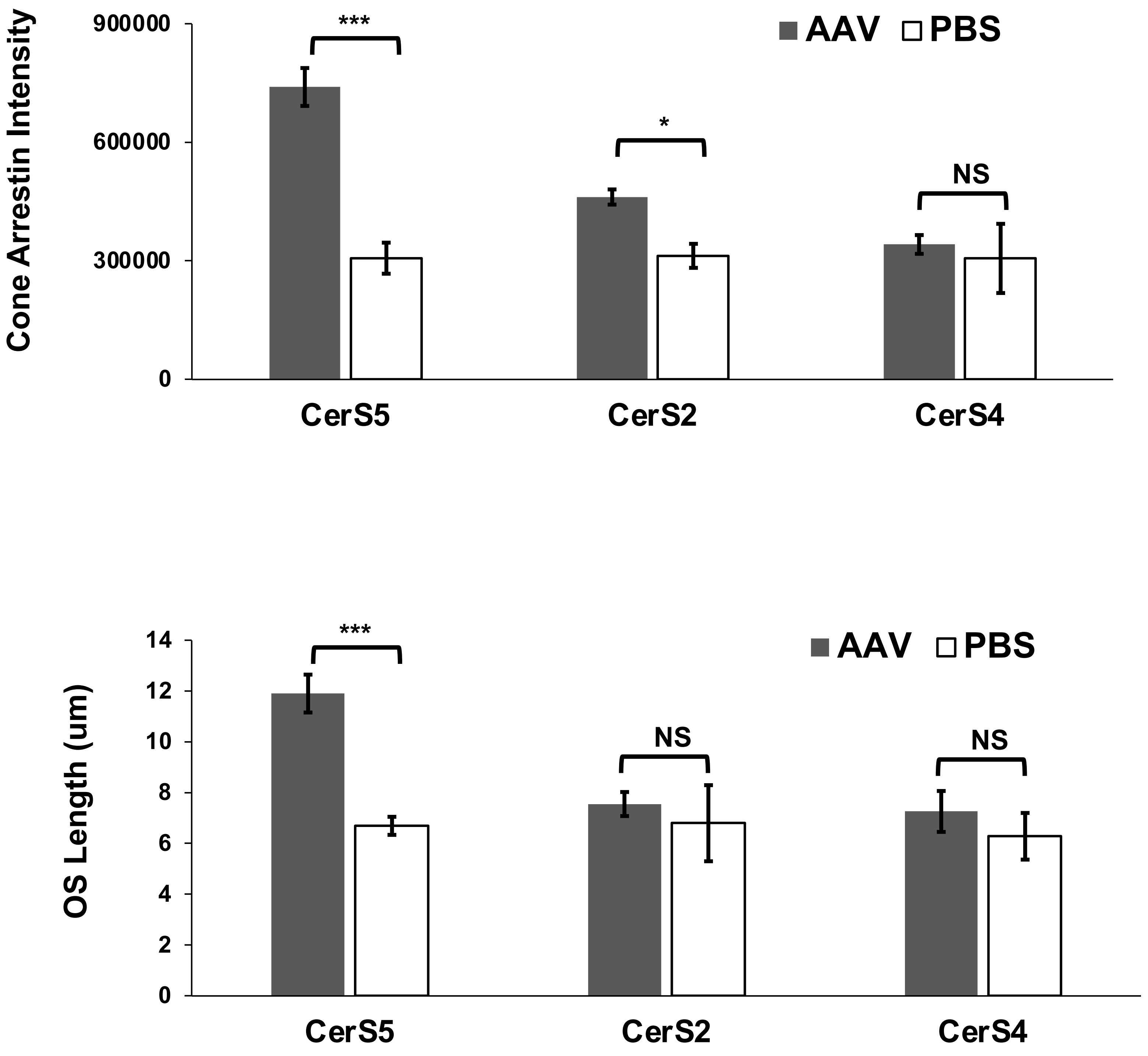

Fig. S4. Quantification results of A) cone arrestin staining intensity and B) the lengths of outer segments of cone cells. Measurements and quantifications were performed in 4 equally spaced regions along the vertical median of the retina (n= 4 for AAV-treated RE and n=4 for PBS-treated LE). Error bars denote the SEM. Student t-tests were performed.
*P < 0.05, **P < 0.01, ***P < 0.001.

Disease Models & Mechanisms • Supplementary information
